# Unveiling *Salmonella* Derby Survival: Stress Responses to Prolonged Hyperosmotic Stress

**DOI:** 10.3390/foods14091440

**Published:** 2025-04-22

**Authors:** Yingting Gong, Xiaoxuan Li, Junying Wang, Yanyan Zhao, Jingnan Meng, Ligong Zhai

**Affiliations:** 1 Food Science and Engineering, Anhui Science and Technology University, Chuzhou 233100, China; 15079552168@163.com (Y.G.); 18956122182@163.com (X.L.); wangjy@ahstu.edu.cn (J.W.); zhaoyanyan@ahstu.edu.cn (Y.Z.); xuanwangmjn@163.com (J.M.); 2 Province Key Laboratory of Functional Agriculture and Functional Food, Anhui Science and Technology University, Chuzhou 239000, China

**Keywords:** foodborne pathogen, processed foods, microbial hazards, osmotic stress tolerance

## Abstract

The traditional marination process enhances food flavor and inhibits microbial growth. However, in hyperosmotic environments, microorganisms can activate stress responses to ensure survival, potentially compromising food safety. This study investigated the osmotolerance mechanisms of *Salmonella* Derby (*S*. Derby) by comparing a wild-type strain (*S*. D-WT) and an osmotolerant strain (*S*. D-OT) under NaCl-induced hyperosmotic stress. Both strains were subjected to 0.85%, 4%, and 16% NaCl for 0, 8, and 16 days, and their growth behavior, membrane integrity, intracellular osmoprotectant content, and transcription of related genes were evaluated. By day 16, both strains showed a growth delay of approximately 3 h. *S*. D-OT maintained better membrane integrity and exhibited higher intracellular levels of osmoprotectants (K⁺, trehalose, and proline), which aligned with the upregulation of the transcriptional levels of *kdpC*, *kuP*, *rpoS*, and *proU*. These findings indicated that *S*. D-OT achieved improved osmotic stress tolerance by regulating osmoprotectant synthesis and maintaining intracellular homeostasis. In contrast, *S*. D-WT displayed greater resistance to multiple antibiotics (gentamicin, ciprofloxacin, trimethoprim-sulfamethoxazole, and chloramphenicol) under 4% and 16% NaCl conditions, which may pose a higher food safety risk. Overall, this study provides insights for improving microbial control strategies in preserved foods and mitigating foodborne disease risks.

## 1. Introduction

*Salmonella* enterica is recognized as the second most prevalent zoonotic pathogen in the European Union (EU) over the past decade and remains a leading cause of foodborne illnesses. In 2023, the EU reported a total of 77,486 confirmed human cases of salmonellosis, reflecting a 16.9% increase compared to 2022 [1]. Clinical manifestations of *Salmonella* infections typically include gastroenteritis, characterized by symptoms such as diarrhoea, fever, vomiting, and abdominal pain [2]. Potential sources of *Salmonella* contamination include contaminated raw meat, added ingredients, processing equipment, reprocessing processes, and the processors themselves [3]. A notable example of a high-risk product is dry fermented sausages, such as salami, which are widely consumed as ready-to-eat products in Europe. These sausages are popular for their distinct fermented flavor and nutrient-rich meat content [4]. However, since traditional salami production does not involve sterilization, the potential presence of *Salmonella* contamination makes it a significant source of foodborne illness [5]. Research has demonstrated that swine herds and ground pork used in sausage manufacturing have a *Salmonella* prevalence of 93.9%, further underscoring the risk of contamination within the supply chain [6]. In 2023, *Salmonella* Derby (*S*. Derby) was one of the predominant *Salmonella* serovars isolated from pigs and pork products across the EU. Specifically, it accounted for 24.8% of the serotyped isolates from pig sources, including both live pigs and pork products [1]. This statistic highlights the elevated risk of *S*. Derby infection in regions where pork is a significant dietary component.

Salt plays a dual role in food production, functioning both as a flavor and texture enhancer, as well as a preservative by reducing water activity, which is crucial for ensuring microbiological safety. In pickled foods, salt is frequently used as an additive to enhance flavor and inhibit microbial growth [7]. However, this may increase the risk of individuals contracting foodborne infections. In hyperosmotic environments, bacteria employ several strategies to manage intracellular osmotic balance. Notably, they utilize a two-component system for potassium (K⁺) regulation and the Trk system to mediate K⁺ accumulation [8]. Additionally, transport proteins such as ProP, ProU, BetT, BetU, OtsA, and OtsB facilitate the uptake of osmoprotectants, including proline, betaine, and trehalose [9]. These regulatory mechanisms increase the intracellular osmotic pressure in bacteria, thereby effectively mitigating the impact of the external hyperosmotic environment and promoting enhanced survival and proliferation. Research has shown that *Salmonella* spp., when exposed to osmotic stress and other environmental challenges, activate various survival mechanisms, including changes in surface structures and the regulation of stress-response genes. For example, a study by Wang et al. demonstrated that *Salmonella* adapted to osmotic stress by upregulating the expression of small non-coding RNAs (sRNAs) [10]. Moreover, *S*. Derby was a prevalent serotype in the pork production chain, exhibiting genetic persistence. Yvon et al. found that *S*. Derby displayed minimal genetic variation from pig carcasses to dry-cured fermented sausage (DFS), suggesting strong adaptability throughout the production process [11]. Therefore, this study selected *S*. Derby as a representative strain to investigate *Salmonella* adaptation strategies under hyperosmotic conditions, providing crucial insights into the behavior of other *Salmonella* strains in similar environments.

To avoid the confounding effects of multiple environmental variables in food matrices that may interact to modulate *Salmonella*’s stress response, this study employed NaCl solutions of varying concentrations to simulate a hyperosmotic environment. The physiological responses, cell membrane integrity, intracellular osmoprotectant levels, and transcription levels of related genes in *Salmonella* Derby were systematically analyzed across different durations of osmotic stress. The findings revealed the survival characteristics of *S*. Derby in hyperosmotic conditions and its regulatory mechanisms for osmotic tolerance. These results provide valuable theoretical support for the prevention and control of microbiological safety in the cured meat products industry and help reduce the risk of foodborne contamination.

## 2. Materials and Methods

### 2.1. Strains and Culture Conditions

The bacterial strains used in this study were the wild-type strain *S*. D-WT (CMCC50719) and an osmotically adapted variant, *S*. D-OT. The *S*. D-OT strain was domesticated in our laboratory [12]. The *S*. D-OT strain was developed from the *S*. D-WT strain through the following adaptation process: Initially, the *S*. D-WT strain was inoculated onto trypticase soy agar (TSA, Beijing Land Bridge Technology Co., Ltd., Beijing, China) containing 4% (*w*/*v*) NaCl and incubated at 37 °C for 24 h. Subsequently, a single colony was transferred to normal trypticase soy broth (TSB, Beijing Land Bridge Technology Co., Ltd., Beijing, China) and incubated at 37 °C with shaking at 180 rpm for 16 h, gradually enhancing the strain’s osmotolerance. This procedure was repeated for a total of 10 cycles, after which the *S*. D-OT strain was successfully obtained. Both strains, *S*. D-WT and *S*. D-OT, were resuscitated on TSA at 37 °C for 24 h. The *S*. D-WT strain was cultured on standard TSA, while the *S*. D-OT strain was propagated on TSA supplemented with 4% (*w*/*v*) NaCl.

### 2.2. Preparation of Hyperosmotic Stress Cells

The two strains were separately cultured in TSB (37 °C, 180 rpm, 16 h). Bacterial cultures (10 mL) of both *S*. D-WT and *S*. D-OT strains were centrifuged at 8000× *g* for 10 min. The supernatant was discarded, and the resulting pellet was washed twice with sterile phosphate-buffered saline (PBS, Sangon Biotech Shanghai Co., Ltd., Shanghai, China) to remove residual medium. After washing, the pellet was resuspended in sterile PBS. Ten milliliters of resuspended bacterial cells were inoculated into 90 mL of sterile saline containing 0.85%, 4%, and 16% (*w*/*v*) NaCl. The mixtures were thoroughly mixed and incubated at 25 °C for 0 days (4 h), 8 days, and 16 days.

### 2.3. Physiological Characterization of S. Derby Under Hyperosmotic Stress

#### 2.3.1. Growth Curves

The growth curves of the two strains were assessed at different NaCl concentrations on days 0, 8, and 16, respectively. Each experiment group was transferred to TSB at a 1:100 dilution ratio and incubated at 37 °C with shaking at 180 rpm/min. The optical density (OD) at 600 nm (OD_600_) was monitored, with the OD value of the sterile culture solution serving as the baseline. Subsequent measurements of OD_600_ were taken hourly, with monitoring continuing for from 12 to 15 h until the cultures reached a stable growth phase.

#### 2.3.2. Viable Cell Count

The viable cell counts of both strains were determined at different NaCl concentrations on days 0, 8, and 16, respectively. Each experiment group was serially diluted in sterile saline using a 10-fold gradient. From each dilution, 0.1 mL of the bacterial suspension was plated onto plate count agar (PCA, Beijing Land Bridge Technology Co., Ltd., Beijing, China) plates and incubated at 37 °C for 24 h. Plates with colony counts ranging from 30 to 300 colonies were selected, and the colony-forming units (CFUs) were counted. The results were expressed as log CFU/mL. By comparing the dynamic changes in viable bacterial counts (log CFU/mL) between the experimental and control groups, the net change in bacterial numbers under stress conditions was calculated as follows: Net Bacterial Count Change (log CFU/mL) = Experimental Group (log CFU/mL) − Control Group (log CFU/mL).

### 2.4. Membrane Integrity of S. Derby Under Hyperosmotic Stress

#### 2.4.1. Cell Membrane Integrity Damage

In this study, propidium iodide (PI; Beijing Solarbio Science & Technology Co., Ltd., Beijing, China), a DNA-binding fluorescent dye, was used to assess the extent of cell membrane damage in *Salmonella* Derby under hyperosmotic stress. This was based on a previously published method with minor modifications [13]. A 1 mL sample from each experimental group was centrifuged at 8000× *g* for 10 min. The supernatant was discarded, and the resulting pellet was washed three times with sterile PBS. The bacterial cells were then resuspended in 1 mL of PI solution (25 μg/mL) for subsequent analysis. The suspension was incubated in the dark at room temperature for 30 min. The microplate reader (Varioskan Flash, Thermo Fisher Scientific, Waltham, MA, USA) was used for fluorescence detection, with the following parameter settings: excitation wavelength at 550 nm, emission wavelength from 620 nm to 700 nm, slit width of 8 nm, and gain value of 80.

#### 2.4.2. Cell Membrane Permeability

Cell membrane permeability and integrity were evaluated by measuring UV absorbance at 260 nm and 280 nm to quantify the leakage of intracellular nucleic acids and proteins, respectively, in *S*. Derby cells subjected to hyperosmotic stress. This method was adapted from a previously published protocol with minor modifications [14]. A 1 mL sample from each experimental group was centrifuged at 8000 rpm for 10 min, and the supernatant was collected. The absorbance at 260 nm and 280 nm was measured using an Ultramicro UV–Vis Spectrophotometer (NV3000C; VASTECH Technology, San Jose, CA, USA). Baseline corrections were applied using the corresponding NaCl solution as a reference.

### 2.5. Cellular Solute Accumulation in S. Derby Under Hyperosmotic Stress

Cell content was determined using previously reported methods with minor modifications [15]. A 40 mL sample from each experimental group was centrifuged at 8000 rpm for 15 min. The supernatant was discarded, and the pellet was washed three times with sterile PBS. Cells from each experimental group were disrupted using an ultrasonic cell crusher (JY99-II.DN; Ningbo Scientz Biotechnology Co., Ltd., Ningbo, China) to release intracellular contents for subsequent analysis. The parameters for ultrasonic treatment were uniformly set as follows: 400 W, duration 3 s, interval 10 s, and a total treatment time of 15 min for cell lysis.

#### 2.5.1. Intracellular Potassium Concentration

**Figure A1:**
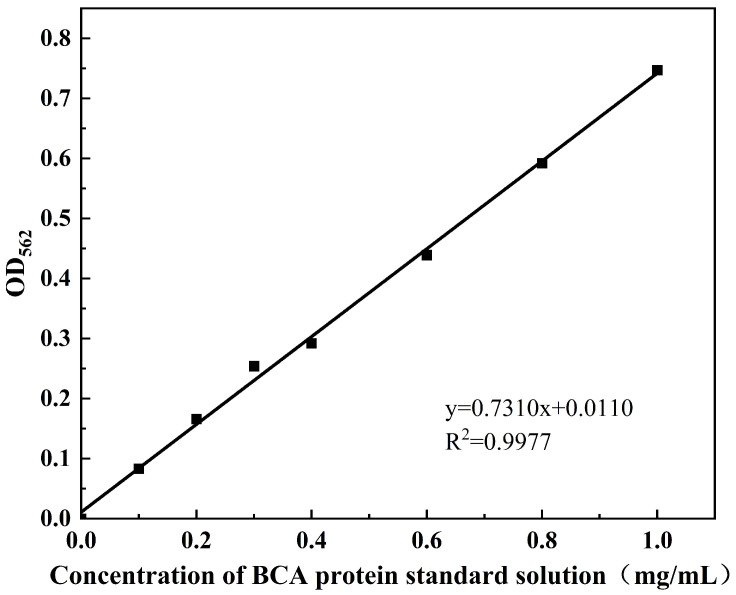
Standard curve of intracellular BCA protein.

**Figure A2:**
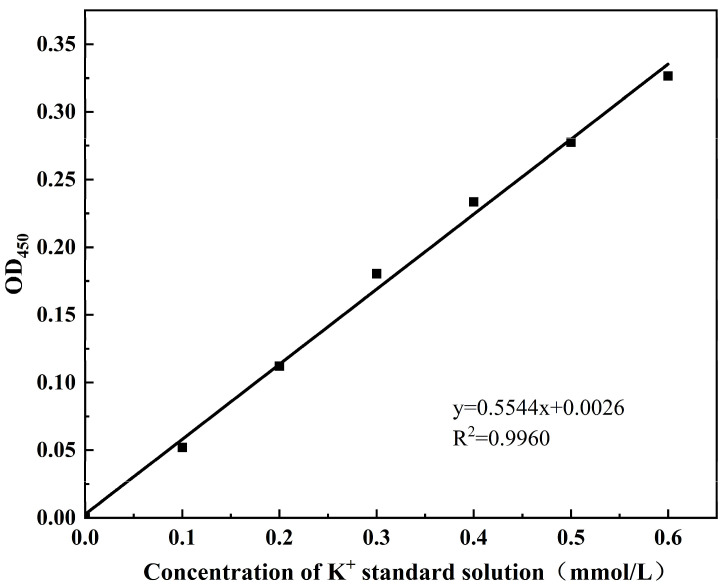
Standard curve of intracellular potassium (K^+^) concentration.

The intracellular Bicinchoninic Acid Assay (BCA) protein concentration (mg/mL) and potassium (K⁺) content (mmol/g protein) were measured according to the protocols of the BCA Protein Colourimetric Assay Kit (Elabscience Biotechnology Co., Ltd., Wuhan, China) and the Potassium (K) Turbidimetric Assay Kit (Elabscience Biotechnology Co., Ltd., Wuhan, China), respectively. The standard curve for intracellular BCA protein was shown in Figure A1, with the corresponding calculation formula provided in Equation (1). The standard curve for intracellular potassium ion (K⁺) concentration is shown in Figure A2, and the calculation formula is given in Equation (2).

BCA protein concentration (mg/mL) = (ΔA_562_ − b)/a × f (1)

where A was the slope of the BCA protein concentration standard curve, b was the BCA protein concentration standard curve intercept, ΔA_562_ represented the sample OD_562_ value minus the blank OD_562_ value, and f was the dilution of the sample prior to addition to the assay system.

K^+^ concentration (mmol/g prot) = (ΔA_450_ − b)/a × 10 × f/C _pr_ (2)

where A was the slope of K^+^ concentration standard curve, b was the K^+^ concentration standard curve intercept, ΔA_450_ represented the sample OD_450_ value minus the blank OD_450_ value, f was the dilution of the sample prior to addition to the assay system, and C _pr_ was the protein concentration of the sample to be measured (g prot/L).

#### 2.5.2. Intracellular Trehalose Concentration

The intracellular trehalose concentration (mg/mg protein) was determined using the Trehalose Content Assay Kit (Beijing Solarbio Science & Technology Co., Ltd., Beijing, China) following the manufacturer’s instructions. The intracellular trehalose content was calculated using the formula provided in Equation (3).

Trehalose concentration (mg/mg prot) = X/C _pr_ (3)

where X (mg/mL) was the concentration of samples determined from trehalose standard curves, and C _pr_ was the protein concentration of the sample to be measured (g prot/L).

#### 2.5.3. Intracellular Betaine Concentration

The intracellular betaine concentration (mg/mL) was determined using the Betaine Content Assay Kit (Shanghai Enzyme-linked Biotechnology Co., Ltd., Shanghai, China) following the manufacturer’s instructions. The betaine content was quantified based on the standard curve provided by the kit, and the corresponding calculation formula was given in Equation (4).

Betaine concentration (mg/mL) = (ΔA_525_ − 0.0057)/1.004 × V_1_/V_2_/C r (4)

where V_1_ was the volume of extract added, V_2_ was the amount of sample added at the extraction stage, ΔA_525_ represented the sample OD_525_ value minus the blank OD_525_ value, and C r was the concentration of multiples of the samples prior to addition to the assay system.

#### 2.5.4. Intracellular Proline Concentration

The intracellular proline concentration (μg/mL) was quantified using the Proline (PRO) Content Assay Kit (Nanjing Jiancheng Bioengineering Institute, Nanjing, China) according to the manufacturer’s instructions. The proline content was determined based on the standard curve provided by the kit, and the corresponding calculation formula is given in Equation (5).

proline concentration (μg/mL) = (ΔA_520_/ΔA_1_) × C/(V_2_/V_1_)/C r (5)

where V_1_ was the volume of extract added, V_2_ was the amount of sample added at the extraction stage, ΔA_520_ represented the sample OD_520_ value minus the blank OD_520_ value, ΔA_1_ represented the standard sample minus the blank OD_520_ value, and C r was the concentration of multiple samples prior to addition to the assay system.

### 2.6. Transcription of Genes in S. Derby Under Hyperosmotic Stress

#### 2.6.1. RNA Extraction and cDNA Synthesis

Total RNA from *S*. D-WT and *S*. D-OT cells was extracted using the Total RNA Extraction Kit (Sangon Biotech, Shanghai, China) according to the manufacturer’s instructions. The extracted RNA was immediately placed on ice to prevent degradation. All subsequent procedures were performed on ice. RNA was then reverse transcribed into cDNA using the AMV First Strand cDNA Synthesis Kit (Sangon Biotech, Shanghai, China) to generate cDNA for subsequent qPCR analysis. The reverse transcription reaction was performed in a Polymerase Chain Reaction (PCR) amplifier (DHG-9030A; Shanghai Sanfa Scientific Instrument Co., Ltd., Shanghai, China) under the following conditions: 42 °C for 60 min, followed by 85 °C for 5 min.

#### 2.6.2. QPCR Analysis

The transcription of potassium-related genes (*ktrB*, *kdpC*, *trkE*, *kuP*), the osmoprotectant gene *proU*, and the stress-responsive gene *rpoS* was analyzed in both *S*. D-WT and *S*. D-OT under different osmotic pressure conditions. Gene transcription levels were measured using Quantitative Real-time PCR (LightCycler^®^ 96 System; F. Hoffmann-La Roche Ltd., Basel, Switzerland). The qPCR reaction mixture was prepared as follows: 2 µL of cDNA, 12.5 µL of TB Green^®^ Premix Ex Taq™ II (Takara Biomedical Technology, Beijing, China), 1 µL of gene-specific upstream and downstream primers (10 μM) each (Table 1), and 8.5 µL of RNase-free ddH_2_O. The thermal cycling conditions were as follows: pre-denaturation at 95 °C for 5 min, followed by 40 cycles of denaturation at 95 °C for 15 s, annealing at 55 °C for 30 s, and extension at 72 °C for 15 s.

#### 2.6.3. Quantification of Relative Gene Transcription

QPCR was employed to analyze the transcriptional levels of the target genes. In the qPCR experiment, the melt curve analysis consistently showed a Tm value within the range of 70–90 °C, with a single peak observed, indicating specific amplification. The *16S rRNA* gene was used as the reference gene, and the relative transcription levels of the target genes were calculated using the 2^^(−ΔΔCt)^ method. The untreated *S*. D-WT strain was used as the baseline for inter-group comparisons. To ensure the reliability and reproducibility of the results, the experiment was conducted with four independent biological replicates, each with three technical replicates. The final results were presented as the mean ± standard deviation (Mean ± SD) of the four biological replicates, accurately reflecting the transcriptional changes of the target genes under different treatment conditions.

### 2.7. Antibiotic Susceptibility Testing of S. Derby Under Hyperosmotic Stress

Antibiotic susceptibility was assessed using the paper diffusion method with the following antibiotics: ceftriaxone (CTR, 30 μg), gentamicin (GEN, 10 μg), imipenem (IPM, 10 μg), minocycline (MI, 30 μg), ciprofloxacin (CIP, 5 μg), trimethoprim-sulfamethoxazole (SXT, 25 μg), and chloramphenicol (C, 30 μg). This method was adapted from a previous study with minor modifications [16]. A total of 100 μL of bacterial suspension from each experimental group was taken and evenly spread on Mueller–Hinton (MH, Beijing Land Bridge Technology Co., Ltd., Beijing, China) agar plates. Following sufficient absorption, antibiotic discs were placed at the center of the plates using sterile tweezers. The plates were incubated at 37 °C for 24 h in a biochemical incubator. The diameters of the inhibition zones (mm) were measured. Interpretation of resistance or susceptibility for each antibiotic was performed based on CLSI criteria, with results classified as susceptible (S), intermediate (I), or resistant (R), as shown in Table 2 [17].

### 2.8. Statistical Analysis

Each experiment was independently performed at least three times, with each repetition carried out in triplicate. Statistical analyses were conducted using one-way analysis of variance (ANOVA), followed by Duncan’s test for multiple comparisons, using IBM SPSS Statistics software (version 26; Armonk, NY, USA). A *p*-value of less than 0.05 was considered statistically significant.

## 3. Results

### 3.1. Physiological Characterization

**Figure 1:**
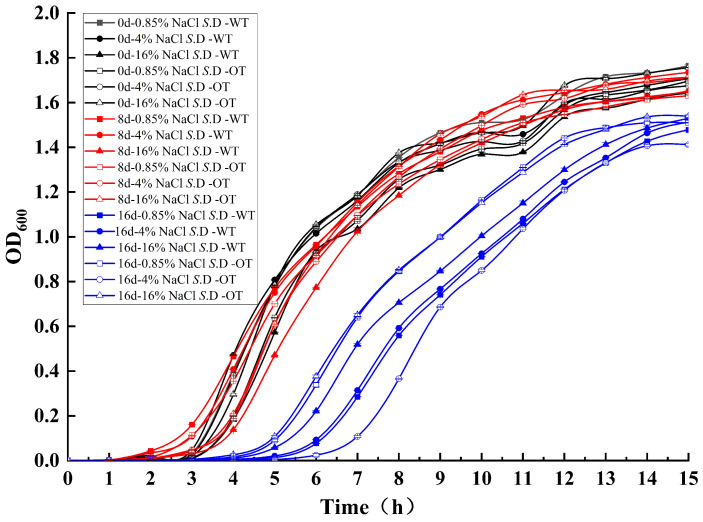
Growth curves of *S*. D-WT and *S*. D-OT under varying NaCl concentrations over different exposure durations.

The cell growth rate is a critical determinant of bacterial adaptation to the environment, influencing both the efficiency of resource utilization and the transcription of metabolic and biosynthetic proteins [18,19]. To assess *S*. Derby adaptability under varying salt concentrations, the growth of each experimental group was analyzed through growth curves in different NaCl solutions (Figure 1). The results indicated small differences between the growth curves of the two strains in the three NaCl solutions at the initial stages of stress (day 0 and day 8), suggesting that both strains exhibited similar growth patterns during short-term exposure. However, by day 16, the growth curves of both strains showed changes: the lag phase was extended from 3 h to 6 h, with an additional lag period of approximately 3 h, and the growth rate decreased. Furthermore, the OD_600_ values of *S*. D-WT and *S*. D-OT at day 16, when they entered the stationary phase, decreased by approximately 0.2 compared to days 0 and 8, indicating a reduction in cell numbers. These findings suggested that the inhibitory effect of the hyperosmotic environment on bacterial growth became more pronounced over time, resulting in prolonged growth retardation, a reduced growth rate, and a decrease in the number of viable cells.

**Figure 2:**
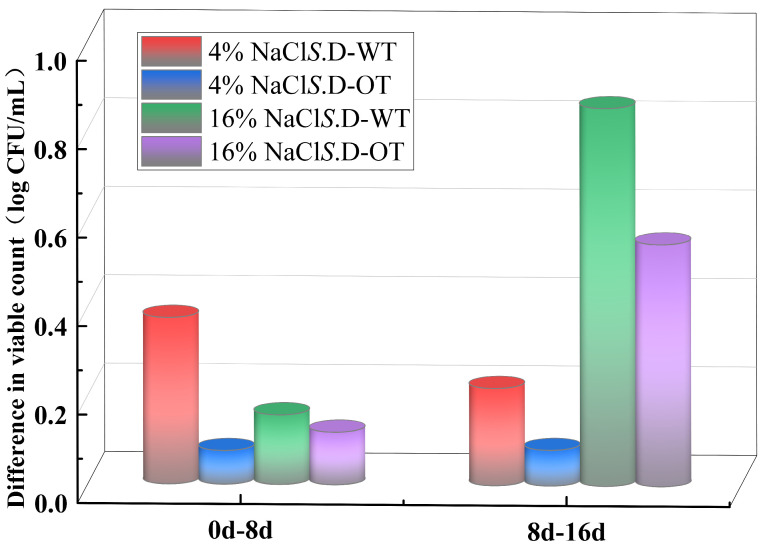
Reduction in viable cell counts of *S*. D-WT and *S*. D-OT at different NaCl concentrations over distinct time intervals (0–8 days, 8–16 days).

In this study, the viability of *S*. Derby under hyperosmotic stress conditions was assessed by comparing the changes in viable cell counts across different time points of exposure. As shown in Figure 2, in a 4% NaCl solution, the viable cell count of *S*. D-WT decreased by 0.38 log CFU/mL during the initial phase of stress (from day 0 to day 8). In comparison, in a 16% NaCl solution, the viable cell count dropped by 0.85 log CFU/mL during the later phase of stress (from day 8 to day 16). In contrast, the viable cell count of *S*. D-OT under the same stress conditions exhibited a slower decline. These findings indicated that the survival ability of *S*. D-OT was greater than that of *S*. D-WT in a hyperosmotic environment. 

### 3.2. Cell Membrane Integrity and Permeability

**Figure 3:**
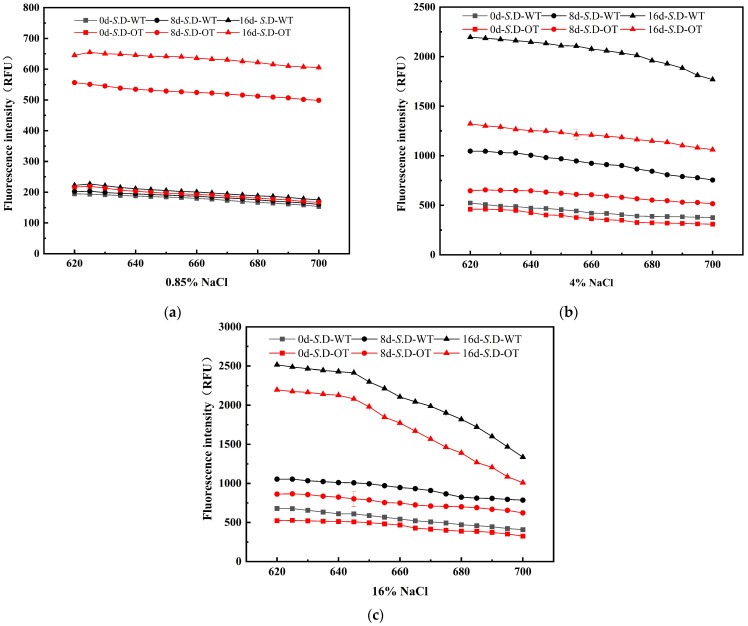
Fluorescence spectra of PI-stained *S*. D-WT and *S*. D-OT cells with membrane damage after 0, 8, and 16 days of exposure to 0.85%, 4%, and 16% NaCl solutions. (**a**) 0.85% NaCl solutions; (**b**) 4% NaCl solutions; (**c**) 16% NaCl solutions.

The cell membrane serves as the external barrier of the cell, and its integrity is essential for maintaining normal physiological functions and phenotypic stability [20]. Upon exposure to exogenous stress, the cell wall and membrane are typically the first structures to experience damage. To assess the differences in cellular phenotypes between the two strains under hyperosmotic stress, the integrity of the cell membrane in *S*. D-WT and *S*. D-OT was analyzed in this study. PI is a small, hydrophilic fluorescent dye commonly used to assess cell membrane integrity. PI cannot penetrate the intact cell membrane of viable cells but can enter cells with compromised membranes, binding to double-stranded DNA and emitting red fluorescence [21]. The intensity of the fluorescence correlates with the degree of membrane damage: higher fluorescence intensity indicates greater membrane permeability and more severe damage. The experimental results demonstrated an increasing trend in fluorescence intensity for both strains under hyperosmotic stress (Figure 3). In a 0.85% NaCl solution, the fluorescence intensity of *S*. D-OT was consistently higher than that of *S*. D-WT, suggesting more significant damage to the cell membrane of *S*. D-OT. In contrast, in both 4% and 16% NaCl solutions, the fluorescence intensity of *S*. D-WT was consistently higher than that of *S*. D-OT, indicating that *S*. D-OT exhibited greater tolerance to these two higher salt concentrations. In conclusion, *S*. D-OT demonstrated superior adaptability compared to *S*. D-WT under hyperosmotic stress conditions, and the damage to the cell membrane of *S*. D-WT became more pronounced with increasing salt concentration.

**Figure 4:**
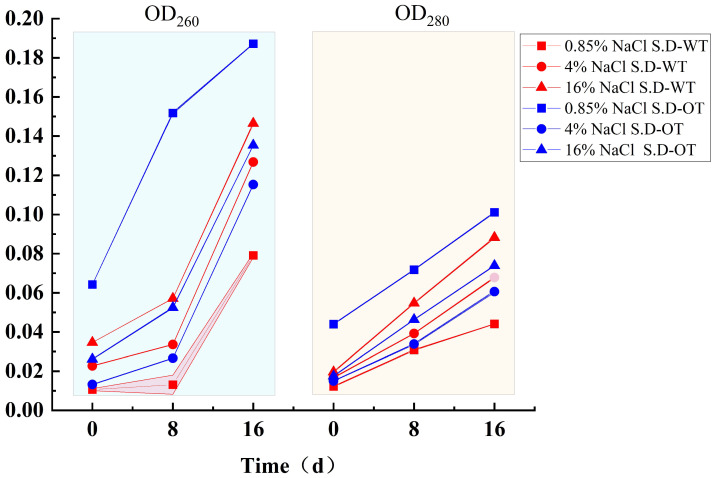
Nucleic acid and protein leakage in *S*. D-WT and *S*. D-OT after 0, 8, and 16 days of exposure to 0.85%, 4%, and 16% NaCl solutions.

When the cell membrane is compromised, it leads not only to the leakage of small molecules but also to the release of larger molecules such as DNA and protein [22]. The extent of nucleic acid and protein leakage can be quantified by measuring the optical densities at 260 nm (OD_260_) and 280 nm (OD_280_), respectively. As shown in Figure 4, the OD_260_ and OD_280_ values for both *S*. Derby strains increased with increasing salt concentration, indicating that both nucleic acid and protein leakage increased, reflecting increased membrane permeability. During hyperosmotic stress treatment, the leakage of nucleic acids and proteins in *S*. D-WT was consistently greater than that in *S*. D-OT over time. Notably, in the 0.85% NaCl solution, *S*. D-OT exhibited higher nucleic acid and protein leakage compared to the other samples. This finding suggested that a low Na⁺ concentration environment was less favorable for the growth and metabolic activities of *S*. D-OT.

### 3.3. Intracellular Osmoprotectant Concentration

Under hyperosmotic stress conditions, *Salmonella* adapts to environmental changes through a variety of strategies, with one key mechanism being the accumulation of intracellular solutes to maintain osmotic pressure homeostasis across the cell membrane [23]. These solutes include K⁺, trehalose, betaine, proline, and other osmoprotectants. In this study, we investigated the molecular mechanisms underlying hyperosmotic tolerance in *Salmonella* by measuring the concentration of intracellular solutes in two *S*. Derby strains exposed to hyperosmotic stress. Additionally, we examined how these mechanisms contribute to their survival strategies in hyperosmotic environments.

**Figure 5:**
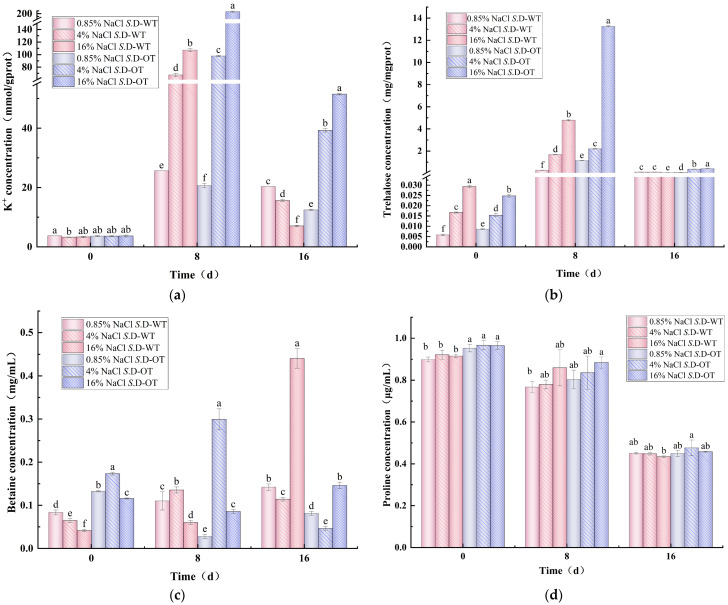
Intracellular solute concentration in *S*. D-WT and *S*. D-OT after 0, 8, and 16 days of exposure to 0.85%, 4%, and 16% NaCl solutions. (**a**) Intracellular K⁺ concentration (mmol/g prot); (**b**) intracellular trehalose concentration (mg/mg prot); (**c**) intracellular betaine concentration (mg/mL); (**d**) intracellular proline concentration (μg/mL). Data were presented as mean ± SD (*n* = 3). Different lowercase letters indicated significant differences among treatment groups (0.85%, 4%, and 16% NaCl solutions) at the same time point (0, 8, and 16 days), as determined by Duncan’s test (*p* < 0.05).

K⁺ homeostasis is essential for bacterial survival and plays crucial roles in osmotic regulation, pH homeostasis, protein synthesis regulation, enzyme activation, and membrane potential maintenance [24]. Under hyperosmotic stress conditions, changes in the intracellular K⁺ content of the two *S*. Derby strains exhibited a trend of initial increase followed by a decrease (Figure 5a). On day 0 of the stress treatment, the intracellular K⁺ content of both strains ranged from 3 to 4 mmol/g prot, with no significant differences observed. However, as the stress period progressed, the intracellular K⁺ content of the *S*. D-WT strain treated with 16% NaCl solution reached 107.13 mmol/g prot on day 8, significantly higher than the 67.51 mmol/g prot observed under 4% NaCl treatment (*p* < 0.05). In contrast, the intracellular K⁺ content of the *S*. D-OT strain treated with 16% NaCl solution was 207.77 mmol/g prot, approximately 2.12 times higher than the 97.81 mmol/g prot in the 4% NaCl solution (*p* < 0.05). These findings suggested that higher salt concentrations significantly promote the accumulation of K⁺ in the *S*. Derby. Furthermore, the intracellular K⁺ content of the *S*. D-OT strain remained consistently higher than that of the *S*. D-WT strain after 8 and 16 days of treatment with 4% and 16% NaCl solutions (*p* < 0.05). This indicated that the *S*. D-OT strain was more efficient at accumulating K⁺ to maintain intracellular homeostasis under hyperosmotic stress, thereby enhancing their ability to survive in hyperosmotic environments.

As an essential osmoprotectant, trehalose played a crucial role in microbial resistance to high-salt stress by regulating cellular osmotic pressure [25]. In this study, the intracellular trehalose content of both *S*. Derby strains exhibited a trend of initial increase followed by a decrease under hyperosmotic stress conditions (Figure 5b). The intracellular trehalose content of the *S*. D-OT strain was consistently higher than that of the *S*. D-WT strain under various NaCl concentrations. On day 8, the trehalose content of both strains was higher than that on day 0, indicating that *S*. Derby accumulates more compatible solutes to maintain cellular turgor pressure under hyperosmotic stress. Notably, on day 8 of 16% NaCl solution, the trehalose content of the *S*. D-OT strain was 13.27 mg/mg prot, significantly higher than the 4.79 mg/mg prot observed in the *S*. D-WT strain (*p* < 0.05) and approximately 2.77 times greater. This suggested that the *S*. D-OT strain had a more robust ability to synthesize trehalose under hyperosmotic stress, thereby enhancing its capacity to adapt to hyperosmotic environments.

Betaine is a potent osmoprotectant commonly found in nature, particularly in microorganisms that thrive in hyperosmotic environments. It can be accumulated intracellularly either through uptake from the external environment or via biosynthesis from its precursor, choline. This osmoprotectant plays a critical role in maintaining cellular integrity under osmotic stress conditions by stabilizing proteins and cellular structures, thereby enhancing microbial survival in challenging environments [26]. Heterotrophic microorganisms such as *Salmonella* cannot directly synthesize betaine from inorganic substances. Instead, they rely on the in vivo oxidation of choline to betaine aldehyde, which is subsequently reduced to produce betaine [27,28]. Under a 16% NaCl solution, the intracellular betaine content of *S*. D-WT gradually increased with prolonged stress exposure, reaching 0.44 mg/mL on day 16 (Figure 5c). In contrast, the intracellular betaine content of the *S*. D-OT was significantly lower, at only 0.15 mg/mL (*p* < 0.05). This suggested that *S*. D-WT produced more betaine on day 16 of high osmotic stress and relied more on betaine as an osmoprotectant in the later stages of the hyperosmotic environment.

It was observed that the activity of proline-degrading enzymes in *Salmonella* was directly inhibited under hyperosmotic stress conditions, leading to little or no degradation of accumulated proline. Consequently, proline levels in the *Salmonella* cytoplasm were not regulated by osmotic stress [29]. The increase in proline content was not a result of endogenous synthesis but rather the result of uptake from the external environment via the ProP and ProU transport systems [30,31]. Figure 5d illustrates the changes in intracellular proline content of the *S*. Derby under hyperosmotic stress over time. The results indicated a decreasing trend in the intracellular proline content of both *S*. Derby strains with prolonged stress exposure. This decrease could be attributed to the continued depletion of proline, which served as a primary source of nitrogen or carbon and was subsequently degraded to glutamate, a key intermediate in essential biochemical reactions that supported bacterial survival [32]. Moreover, the intracellular proline content of the *S*. D-OT strain was consistently higher than that of the *S*. D-WT strain.

### 3.4. Gene Transcription Levels

**Figure 6:**
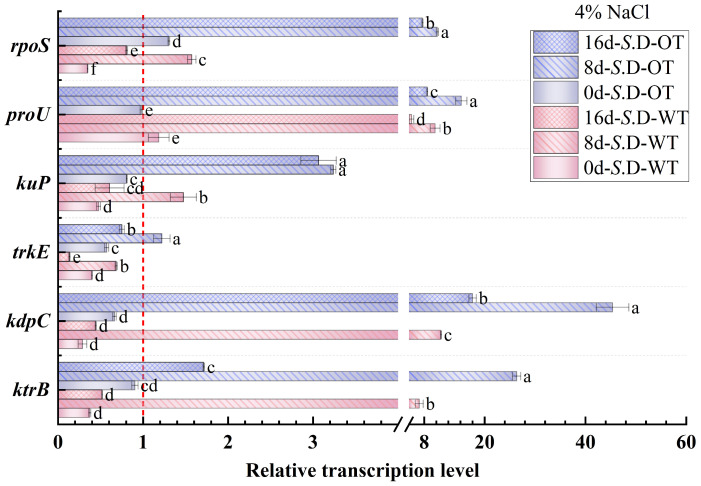
Gene transcription levels of *S*. D-WT and *S*. D-OT during 4% NaCl exposure (0, 8, and 16 days) are presented. Data were presented as mean ± SD from four biological replicates (*n* = 4). Relative transcription levels were calculated using the 2^^(−ΔΔCt)^ method with the untreated *S*. D-WT strain as the calibrator. Values >1, <1, and =1 indicated upregulation, downregulation, and no change, respectively. Different lowercase letters indicated statistically significant differences (*p* < 0.05) in transcript fold-changes among experimental groups as determined by Duncan’s multiple range test.

**Figure 7:**
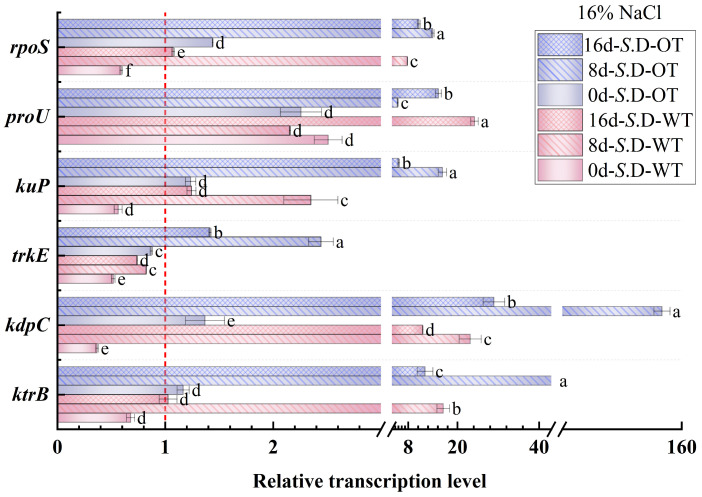
Gene transcription levels of *S*. D-WT and *S*. D-OT during 16% NaCl exposure (0, 8, and 16 days) are presented. Data were presented as mean ± SD from four biological replicates (*n* = 4). Relative transcription levels were calculated using the 2^^(−ΔΔCt)^ method with the untreated *S*. D-WT strain as the calibrator. Values >1, <1, and =1 indicated upregulation, downregulation, and no change, respectively. Different lowercase letters indicated statistically significant differences (*p* < 0.05) in transcript fold-changes among experimental groups as determined by Duncan’s multiple range test.

Gene regulation is a key mechanism underlying bacterial adaptation to environmental changes and the transcription of specific phenotypes [33]. In this study, we investigated the changes in the transcription levels of genes involved in osmotic pressure regulation in *S*. Derby under long-term hyperosmotic environmental stress to reveal the genetic variations and regulatory mechanisms associated with differences in hyperosmotic tolerance. Figure 6 and Figure 7 show the relative transcription levels of the *ktrB*, *kdpC*, *trkE*, *kuP*, *proU*, and *rpoS* genes in the two *S*. Derby strains after prolonged exposure to 4% NaCl and 16% NaCl solutions, respectively. The results indicated that the genes associated with K⁺ transport (*ktrB*, *kdpC*, and *kuP*) exhibited upregulated transcription by day 8 in both the 4% NaCl and 16% NaCl solutions for both strains. Moreover, the transcription levels of these genes were higher in the *S*. D-OT strain compared to the *S*. D-WT strain. These findings aligned with the previously observed trend in intracellular K⁺ content, supporting the enhanced osmotic tolerance of the *S*. D-OT strain. Notably, at day 8, in the 16% NaCl solution, the transcription levels of *kdpC* and *kuP* in *S*. D-OT were 153.6-fold and 16.34-fold higher than the control, respectively, significantly exceeding the transcription levels observed in *S*. D-WT (23.14-fold and 2.35-fold, respectively) (*p* < 0.05). The high transcription of the *kdpC* gene was likely associated with the function of the KdpFABC complex, a high-affinity potassium-ion transporter system that efficiently uptakes potassium ions in low potassium environments, thus contributing to the strain’s ability to maintain potassium homeostasis under hyperosmotic stress [34].

The RpoS factor plays a crucial role in bacterial responses to environmental stresses such as hyperosmolarity and oxidation [35]. The *rpoS* gene not only regulates gene transcription under steady-state conditions but also activates the transcription of specific small RNAs (sRNAs) in response to a variety of environmental stresses, thereby promoting the translation of RpoS [36]. In this study, the *rpoS* gene showed upregulated transcription in both strains by day 8. Under 4% NaCl stress, the *rpoS* transcription level in *S. D-WT* was 1.57-fold compared to the control. Exposure to 16% NaCl induced upregulation to 7.79-fold, suggesting that hyperosmotic stress strongly induced the transcription of the *rpoS* gene. Additionally, the ProU system, an important transport system for proline and betaine in *Salmonella*, is involved in the transport of osmoprotectants. Under 4% NaCl conditions, both strains exhibited upregulation of *proU* transcription by day 8, with *S*. D-WT showing a 15.38-fold and *S*. D-OT a 10.18-fold transcription level compared to the control. Exposure to 16% NaCl resulted in sustained upregulation of *proU* in both strains across all time points (days 0, 8, and 16). Notably, by day 16 under 16% NaCl conditions, the *proU* transcript level in *S*. D-WT reached 24.19-fold of the control, significantly higher than the 15.36-fold induction observed in *S*. D-OT (*p* < 0.05). In summary, the differences in hyperosmotic tolerance among *S*. Derby strains were closely related to the regulation of osmolarity-related gene transcription. The *S*. D-OT strain enhanced tolerance to hyperosmotic stress by regulating the transcription of these key genes more efficiently, thus demonstrating a stronger adaptive capacity.

### 3.5. Antibiotic Susceptibility

Bacterial resistance not only increases the risk to food safety but is also transmitted to humans through the food chain, contributing to the spread of resistance within the population [37]. The paper diffusion method, a widely used bacterial resistance detection technique, determines bacterial susceptibility by measuring the inhibitory effects of antimicrobial agents on bacterial growth [38]. The results of the paper diffusion method were shown in Table 3, which illustrated the antibiotic susceptibility of the two strains after treatment with 4% and 16% NaCl solutions. The experimental results indicated that both strains exhibited different antibiotic sensitivities after prolonged exposure to hyperosmotic stress conditions. The *S*. D-WT strain showed a shift in its sensitivity to IPM from type I to type S, following treatment with 4% and 16% NaCl solutions for 16 days. Similarly, the *S*. D-OT strain demonstrated a change in its sensitivity to GEN from I to S. Additionally, after 16 days of exposure to 4% NaCl solution, the *S*. D-WT strain showed a shift in its sensitivity to CTR from I to S. These findings suggested, that over time, the antibiotic sensitivity of both strains increased, while antibiotic resistance decreased. Under prolonged hyperosmotic stress, mutations in bacterial porins likely facilitated the efficient entry of hydrophilic compounds, such as β-lactam antibiotics (e.g., CTR) and aminoglycosides (e.g., GEN), resulting in reduced resistance to these antibiotics [39]. Under both 4% and 16% NaCl conditions, the *S*. D-WT strain consistently exhibited R to CIP. However, the *S*. D-OT strain showed S to CIP under 4% NaCl stress, and, after 16 days of exposure to 16% NaCl, the *S*. D-OT strain’s sensitivity to CIP shifted from I to S. These results suggested that *S*. D-WT exhibited higher resistance to CIP. Under extreme environmental conditions, environmental stressors can induce the overexpression of efflux pumps, such as MdtEF-TolC and AcrAB-TolC, thereby promoting the efflux of ciprofloxacin. The *S*. D-WT strain may rely more heavily on efflux pumps to alleviate osmotic stress due to its weaker osmoregulatory capacity, resulting in higher resistance to CIP [40].

## 4. Discussion

In hyperosmotic environments, bacteria can maintain intracellular stability by synthesizing protective substances and regulating osmotic pressure through mechanisms that support cell growth and division [41]. However, when hyperosmotic stress surpasses the cell’s adaptive threshold, the accumulation of intracellular ions and biomolecules can lead to DNA damage, disrupted cellular signal transduction, and, ultimately, cell death [19]. In this study, the decrease in cell numbers in *S*. D-OT was smaller across various stress durations, suggesting that *S*. D-OT exhibits a higher tolerance to hyperosmotic stress.

The integrity of the cell membrane is vital for bacteria to cope with hyperosmotic stress. The cell membrane, primarily composed of a phospholipid bilayer, experiences increased tension due to the efflux of intracellular water under hyperosmotic conditions. This change in membrane tension can lead to rearrangement of the phospholipid molecules, thereby enhancing membrane permeability. Such increased permeability disrupts the integrity of the membrane and can induce necrosis or apoptosis [42]. In this study, the cell membrane permeability and PI staining fluorescence intensity of the *S*. D-WT strain were higher than those of the *S*. D-OT strain, indicating that *S*. D-OT possesses a more stable membrane structure and a more effective stress-response mechanism under hyperosmotic conditions. Interestingly, in low-concentration NaCl solutions, the fluorescence intensity and cell membrane permeability of *S*. D-OT were higher than those of *S*. D-WT. This could be attributed to the fact that *S*. D-OT, having adapted to a hyperosmotic environment, relied on a high concentration of Na⁺ to maintain its cell wall structure and function. In contrast, in a low-salt environment, the stability of its cell membrane may have been compromised [43].

Under hyperosmotic stress, *Salmonella* initially mitigates the immediate effects of osmotic pressure changes on the cell membrane by regulating K⁺ concentration. Specifically, the KdpD/KdpE two-component system elevates intracellular K⁺ concentration in response to osmotic stress by regulating the transcription of the KdpFABC complex [44]. In this study, both the intracellular K⁺ concentration and the transcription of K⁺ transport-related genes (*kdpC* and *kuP*) were higher in the *S*. D-OT strain compared to *S*. D-WT, suggesting that *S*. D-OT is more proficient in K⁺ regulation. As the duration of stress increased, *Salmonella* cells further activated the synthesis or uptake mechanisms of osmoprotectants, such as trehalose, proline, and betaine, to enhance osmotic regulation. This was consistent with the findings of Hermann Rath et al. [45]. Notably, under hyperosmotic stress conditions, the intracellular levels of trehalose and proline in the *S*. D-OT strain were higher than those in the *S*. D-WT strain. However, by day 16 post-stress, the intracellular betaine levels in the *S*. D-WT strain were higher than those in the *S*. D-OT strain, indicating that the two strains adopted different strategies for osmotic regulation. Furthermore, in this study, the stress response regulator *rpoS* was upregulated in the *S*. D-OT strain after exposure to 4% NaCl and 16% NaCl hyperosmotic stress, a pattern that aligned with the significant upregulation of *rpoS* (>50-fold) observed in *Salmonella enterica* cells following desiccation adaptation and storage at 4 °C for 2 days [46].

Antibiotic resistance in *Salmonella* is a significant global public health concern. Resistant strains can be transmitted to humans through the food chain, increasing healthcare costs and mortality [47]. Studies have demonstrated that *Salmonella* has evolved highly complex mechanisms, including sensors/receptors, signaling systems, and enzymes/transcription factors, to ensure its tolerance and survival under various harsh environmental conditions. This enhanced stress tolerance not only increases *Salmonella*’s pathogenicity but also compromises the safety of dry-cured meats by sheltering pre-exposed, stress-injured bacterial cells, which later become more virulent [48]. Furthermore, research by Rafaela Martins Morasi et al. has highlighted the high prevalence of virulence gene expression within the *Salmonella* genus. By establishing comparative epidemiological parameters for the pathogen’s potential over time, this study revealed that multidrug-resistant *Salmonella* isolates exhibit enhanced resistance to certain antimicrobial agents and increased expression of genes encoding virulence factors, thereby significantly boosting their pathogenic potential. This may pose a risk to human health through the food chain [49]. In this study, the *S*. D-WT strain exhibited resistance phenotypes to antibiotics such as GEN, CIP, SXT, and C under osmotic pressure conditions of 4% and 16% NaCl solutions. These findings suggested that *Salmonella* could maintain its antibiotic resistance even under high-salt environmental stress, thereby presenting greater challenges for food safety management.

## 5. Conclusions

In this study, the regulatory mechanisms governing the survival of two *S*. Derby strains under hyperosmotic stress were investigated by comparing the survival rates of *S*. D-WT and *S*. D-OT strains exposed to prolonged stress in NaCl solutions of varying concentrations. The findings revealed that the *S*. D-OT exhibited greater cell membrane integrity and selective permeability compared to the *S*. D-WT strain under hyperosmotic stress conditions. Furthermore, genes involved in K^+^ transport (*kdpC*, *kuP*), stress-related (*rpoS*), and osmoprotectants (*proU*) were upregulated in the *S*. D-OT strain, a pattern consistent with the increased intracellular levels of potassium and osmoprotectants (trehalose, proline) observed in the *S*. D-OT. These results underscore the superior capacity of the *S*. D-OT to endure hyperosmotic stress. Interestingly, in a 0.85% NaCl solution, *S*. D-WT demonstrated higher metabolic activity than the *S*. D-OT strain. Moreover, *S*. D-WT exhibited higher resistance to antibiotics (CIP, GEN, SXT, and C) than the *S*. D-OT strain when subjected to 4% and 16% NaCl solutions, thereby posing an increased risk to food safety. This study enhances the understanding of *S*. Derby viability in preserved foods and its mechanisms of differential hyperosmotic tolerance. The findings provide a scientific basis for the food industry to better control the survival of *S*. Derby in food products, thereby reducing the risk of foodborne illnesses.

## Figures and Tables

**Table 1:** Primers for gene transcription analyzed in research.

Gene Function	Gene Name	Primer Sequence (5′→3′)	Amplicon Sizes (bp)	Primer Source
Reference gene	*16S rRNA*	F:CTCTTGCCATCAGATGTGCC R:TTCTTCATACACGCGGCATG	201	Design of this study
K^+^ transport system	*ktrB*	F:GCGATACCGGACTTAGTTTG R:CCATTTTCGACCTGATTTC	697	Design of this study
	*kdpC*	F:ATGATCGGTTTACGTCCTGC R:CATTTCGCTCTGTGTCCCTG	584	Design of this study
	*trkE*	F:CCACTACTCTGGTGGGGC R:ACCGCCAAACCATTGCAG	77	Design of this study
	*kuP*	F:TGGTATTGCCGTCGTTAG R:CAGGAATGCGATGAGGAT	467	Design of this study
Osmoprotectant transport system	*proU*	F:TTACGCTCACGCCTACCC R:GGCAATCCTGTCGCCAAT	241	Design of this study
Stress response regulator	*rpoS*	F:GGAACCCAGTGATAACGAC R:CAGTCCACGATTGCCATAA	244	Design of this study

**Table 2:** Criteria for susceptibility testing determination.

Antibiotic Categories	Antibiotic Name	Circle of Inhibition Judgement Standard (mm)		
		R	I	S
β-lactam	ceftriaxone (CTR) imipenem (IPM)	≤19	20–22	≥23
Aminoglycoside	gentamicin (GEN)	≤12	13–14	≥15
Tetracycline	minocycline (MI)	≤14	15–18	≥19
Quinolone	ciprofloxacin (CIP)	≤20	21–30	≥31
Sulphonamide	trimethoprim-sulfamethoxazole (SXT)	≤10	11–15	≥16
Chloramphenicol	chloramphenicol (C)	≤12	13–17	≥18

**Table 3:** Antibiotic resistance analysis of *S*. D-WT and *S*. D-OT strains under NaCl stress (4% and 16% NaCl solutions) for 0, 8, and 16 days.

Sample	Circle of Inhibition (mm)						
	CTR	GEN	IPM	MI	CIP	SXT	C
0d-4%NaCl *S*. D-WT	20.73 (I)	7.80 (R)	21.97 (I)	15.29 (I)	9.92 (R)	5.98 (R)	6.04 (R)
8d-4%NaCl *S*. D-WT	29.05 (S)	8.01 (R)	26.3 (S)	8.22 (R)	14.65 (R)	5.61 (R)	5.60 (R)
16d-4%NaCl *S*. D-WT	31.75 (S)	8.84 (R)	28.31 (S)	12.76 (R)	17.94 (R)	5.19 (R)	6.30 (R)
0d-16%NaCl *S*. D-WT	23.58 (S)	6.63 (R)	20.91 (I)	17.4 (I)	13.65 (R)	5.92 (R)	5.89 (R)
8d-16%NaCl *S*. D-WT	31.04 (S)	6.73 (R)	28.75 (S)	11.90 (R)	14.92 (R)	5.60 (R)	5.53 (R)
16d-16%NaCl *S*. D-WT	31.54 (S)	11.37 (R)	32.49 (S)	13.28 (R)	15.28 (R)	6.13 (R)	7.56 (R)
0d-4%NaCl *S*. D-OT	28.80 (S)	13.77 (I)	23.21 (S)	19.74 (S)	31.30 (S)	21.64 (S)	29.31 (S)
8d-4%NaCl *S*. D-OT	30.62 (S)	24.23 (S)	26.82 (S)	15.07 (I)	34.86 (S)	23.92 (S)	25.47 (S)
16d-4%NaCl *S*. D-OT	41.61 (S)	36.59 (S)	25.16 (S)	19.19 (S)	41.38 (S)	19.50 (S)	31.75 (S)
0d-16%NaCl *S*. D-OT	23.13 (S)	13.83 (I)	21.04 (I)	16.69 (I)	27.78 (I)	22.29 (S)	21.51 (S)
8d-16%NaCl *S*. D-OT	32.51 (S)	28.31 (S)	27.21 (S)	12.68 (R)	38.73 (S)	26.55 (S)	30.39 (S)
16d-16%NaCl *S*. D-OT	39.19 (S)	33.21 (S)	23.61 (S)	15.82 (I)	42.17 (S)	26.58 (S)	30.90 (S)

S: Susceptible; I: Intermediate; R: Resistant. Antibiotics ceftriaxone (CTR), gentamicin (GEN), imipenem (IPM), minocycline (MI), ciprofloxacin (CIP), trimethoprim-sulfamethoxazole (SXT), and chloramphenicol (C).

## Data Availability

The original contributions presented in this study are included in the article; further inquiries can be directed to the corresponding author.

## Abbreviations

The following abbreviations are used in this manuscript:

*S*. Derby	*Salmonella* Derby
EU	European Union
sRNAs	small non-coding RNAs
DFS	dry-cured fermented sausage
TSA	Trypticase Soy Agar
TSB	Trypticase Soy Broth
PBS	Phosphate-buffered Saline
PCA	Plate Count Agar
BCA	Bicinchoninic Acid Assay
MH	Mueller-Hinton
CFU	Colony-forming units
PI	Propidium Iodide
PCR	Polymerase Chain Reaction
QPCR	Quantitative Real-time PCR
CLSI	Clinical and Laboratory Standards Institute
CTR	Ceftriaxone
GEN	Gentamicin
IPM	Imipenem
MI	Minocycline
CIP	Ciprofloxacin
SXT	Trimethoprim-sulfamethoxazole
C	Chloramphenicol
